# Anti‐EF‐Tu IgG titers increase with age and may contribute to protection against the respiratory pathogen *Haemophilus influenzae*

**DOI:** 10.1002/eji.201847871

**Published:** 2019-01-03

**Authors:** Oskar Thofte, Ravinder Kaur, Yu‐Ching Su, Marta Brant, Anna Rudin, Derek Hood, Kristian Riesbeck

**Affiliations:** ^1^ Clinical Microbiology Department of Translational Medicine Faculty of Medicine Lund University Malmö Sweden; ^2^ Center for Infectious Diseases and Immunology Rochester General Hospital Research Institute Rochester NY; ^3^ Department of Immunology Sahlgrenska University Hospital University of Gothenburg Gothenburg Sweden; ^4^ Mammalian Genetics Unit MRC Harwell Institute Harwell Science & Innovation Campus Oxfordshire UK

**Keywords:** Bacterial infections, Chronic obstructive pulmonary disease, Epitope mapping, Mucosal immunity, Surface antigen

## Abstract

Non‐typeable *Haemophilus influenzae* (NTHi) is a pathogen that commonly colonizes the nasopharynx of preschool children, causing opportunistic infections including acute otitis media (AOM). Patients suffering from chronic obstructive pulmonary disease (COPD) are persistently colonized with NTHi and occasionally suffer from exacerbations by the bacterium leading to increased morbidity. Elongation‐factor thermo unstable (EF‐Tu), a protein critical for bacterial protein synthesis, has been found to moonlight on the surface of several bacteria. Here, we show that antibodies against NTHi EF‐Tu were present in children already at 18 months of age, and that the IgG antibody titers increased with age. Children harboring NTHi in the nasopharynx also displayed significantly higher IgG concentrations. Interestingly, children suffering from AOM had significantly higher anti‐EF‐Tu IgG levels when NTHi was the causative agent. Human sera recognized mainly the central and C‐terminal part of the EF‐Tu molecule and peptide‐based epitope mapping confirmed similar binding patterns for sera from humans and immunized mice. Immunization of BALB/c and otitis‐prone Junbo (C3H/HeH) mice promoted lower infection rates in the nasopharynx and middle ear, respectively. In conclusion, our results suggest that IgG directed against NTHi EF‐Tu may play an important role in the host immune response against NTHi.

## Introduction

The Gram‐negative bacterium *Haemophilus influenzae* is subdivided into encapsulated serotypes a–f that harbor a polysaccharide capsule, and the unencapsulated non‐typeable *H. influenzae* (NTHi). Since the introduction of a capsule‐based vaccine against *H. influenzae* type b (Hib) in the 1990s, NTHi has become the most common *H. influenzae* causing human disease. NTHi often resides asymptomatically in preschool children, and mainly causes opportunistic upper respiratory tract infections, including sinusitis and acute otitis media (AOM) 1. Patients suffering from chronic obstructive pulmonary disease (COPD) are frequently colonized with NTHi, leading to exacerbations and increased morbidity 2. NTHi can also cause invasive disease, but is mainly isolated in immuncompromised hosts or patients with comorbidities 3. Healthy adults, however, may also be infected with more virulent strains resulting in pneumonia and sinusitis 4. A vaccine against NTHi is sought for in order to prevent disease of individuals at risk, and in recent years several vaccine antigens have been defined 5.

From a microbe´s point‐of‐view, several steps, including adhesion to the epithelium and the extracellular matrix (ECM), evasion of the innate immunity, and internalization into epithelial cells, are important for effective colonization and subsequent infection. NTHi is equipped with a plethora of multifunctional virulence factors, and some of these have also been suggested as vaccine candidates 6, 7. Protein D and F are examples of two NTHi proteins that can be found at the surface of virtually all NTHi 8, 9, 10. Protein D has glycerophosphodiesterase activity ensuring a constant essential supply of phosphorylcholine obtained from epithelial cells 11. In contrast, Protein F, in addition to mediating NTHi attachment, interacts with the ECM proteins laminin and vitronectin, the latter of which inhibits the terminal pathway of complement activation 9. Immunocompetent adult individuals carry IgG against Protein D and F 5, 11 and specific antibodies against Protein D have been found to contribute significantly to bactericidal activity in sera obtained from small children 12. Moreover, immunization of children with Protein D may elicit an immune response that mediates partial protection against NTHi‐dependent AOM 13.

In contrast to eukaryotic cells, many prokaryotes have small genomes and consequently limited number of proteins, and therefore, also utilize intracellular proteins for other additional functions, hence, the designation "moonlighting" proteins. A vivid example is elongation factor thermo unstable (EF‐Tu) that is an essential bacterial protein that constitutes up to 5% of the total cell content 14. In *Escherichia coli*, the genes *tufA* and *tufB* encode 40‐ to 45‐kDa EF‐Tu proteins, each containing three structural domains varying only in their C‐termini 15. EF‐Tu binds various guanosine‐containing polyphosphates, and functions in polypeptide elongation with aminoacyl transfer RNAs and guanosine triphosphate. Early studies have shown that EF‐Tu is surface‐exposed in *E. coli* 14. Subsequent investigations demonstrated that EF‐Tu is located at the surface of several other bacterial species including the Gram‐negative *Acinetobacter baumannii*, *Borrelia burgdorferi*, and *Pseudomonas aeruginosa* in addition to the Gram‐positive *Staphylococcus aureus* and *Streptococcus pneumoniae* as well as *Mycoplasma pneumoniae* 16, 17, 18, 19.

In the current study, we hypothesized that an adaptive immune response against NTHi EF‐Tu would induce protection from upper respiratory tract infection caused by NTHi. We found that IgG against EF‐Tu was present in children already at 18 months of age, increased by age, and could be associated with NTHi carriage during early childhood. Human sera and sera from mice immunized with EF‐Tu recognized similar regions on the EF‐Tu molecule as revealed by peptide mapping, and mice were partially protected upon challenge with NTHi. In summary, EF‐Tu is an important target for the adaptive immune defense conferring host protection against NTHi.

## Results

### The adaptive immune response against EF‐Tu begins early in life, and IgG titers increase with age

**Figure 1:**
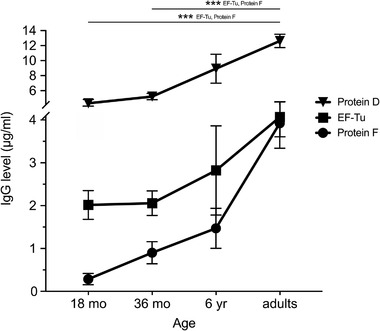
Anti‐EF‐Tu IgG titers and age. IgG levels in plasma from children aged 18 months (*n = *89), 36 months (*n = *92), and 6 years (*n = *23) and sera from adults (*n = *72) directed against EF‐Tu, Protein F, and Protein D were assayed. Data are shown as mean values of triplicate measurements obtained from all individuals in each age group ± SEM. Plasma or serum samples were analyzed once for each individual. ***p < 0.001. Statistical significance was calculated using a one‐way ANOVA with a Bonferroni post hoc test.

To assess whether IgG antibodies against EF‐Tu appears in young and adult humans upon natural exposure to respiratory pathogens, sets of different age‐matched sera were collected from preschool children and healthy adult blood donors. Antibody levels against NTHi proteins were measured by ELISA (Fig. 1). IgG against EF‐Tu was produced at a detectable level at 18 months of age, with the antibody titers significantly increasing in adults. Anti‐EF‐Tu IgG concentrations increased twofold and 1.5‐fold in adults compared to 18‐month‐old and 6‐year‐old children, respectively.

Considering the high level of EF‐Tu sequence conservation between bacterial species 16, 20, the increased concentrations of anti‐EF‐Tu IgG may result not only from NTHi infection, but also from exposure to non‐NTHi bacterial species. For comparison, we, therefore, also analyzed IgG directed against NTHi surface Protein D and F 9, 11 (Fig. 1). Antibody titers increased with age, corresponding with anti‐EF‐Tu, and reached highest levels in adults. IgG concentrations against Protein D and F were three‐ and tenfold higher, respectively, in adults than in 18‐month‐old children. These results thus demonstrate that IgG against NTHi EF‐Tu and two other NTHi antigens increase with age.

### Acute otitis media in children is associated with increased concentrations of anti‐EF‐Tu IgG

**Figure 2:**
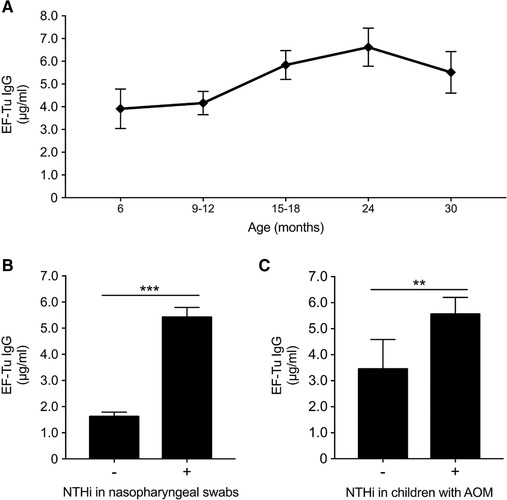
AOM and NTHi infections in children and titers of IgG directed against EF‐Tu. (A) Anti‐EF‐Tu IgG in sera (*n = *135) from a cohort of children (age range 6–30 months) with a nasopharyngeal swab positive for NTHi. (B) Levels of IgG directed against EF‐Tu in children (*n = *97) with NTHi culture‐positive nasopharyngeal swabs and culture‐negative controls (*n = *41). (C) Anti‐EF‐Tu IgG levels in children (*n = *39) with AOM and confirmed NTHi infection relative to children with AOM without previous history of NTHi infection (*n = *19). Data are shown as mean ± SEM and represent measurement of technical triplicates for each serum. All samples were analyzed once. ***p < 0.001; **p < 0.01. Statistical significance was calculated using a one‐way ANOVA with a Bonferroni post hoc test (A), the Student's *t*‐test (B), or Mann–Whitney test (C).

We next assessed whether acute otitis media (AOM) episodes and/or NTHi infection would affect the levels of IgG directed against NTHi EF‐Tu. An experimental cohort comprising young children aged 6 to 30 months that had nasopharyngeal cultures positive for NTHi exhibited elevated levels of IgG directed against NTHi EF‐Tu, increasing significantly with age (Fig. 2A). In comparison with NTHi culture‐negative children, this group had 3.3‐fold more anti‐EF‐Tu IgG (Fig. 2B). From this particular cohort, plasma samples were also selected from children who were suffering from AOM. Cultures were sampled from the middle ear fluid, collected by tympanocentesis during an AOM infection. In this group, we found significantly higher anti‐EF‐Tu IgG titers in patients with a concurrent positive NTHi culture (Fig. 2C). These data suggested that bacterial upper respiratory tract infections, in particular those caused by NTHi, trigger a significant adaptive immune response resulting in more anti‐EF‐Tu IgG.

### Human antibodies recognize the central and C‐terminal regions of NTHi EF‐Tu

**Figure 3:**
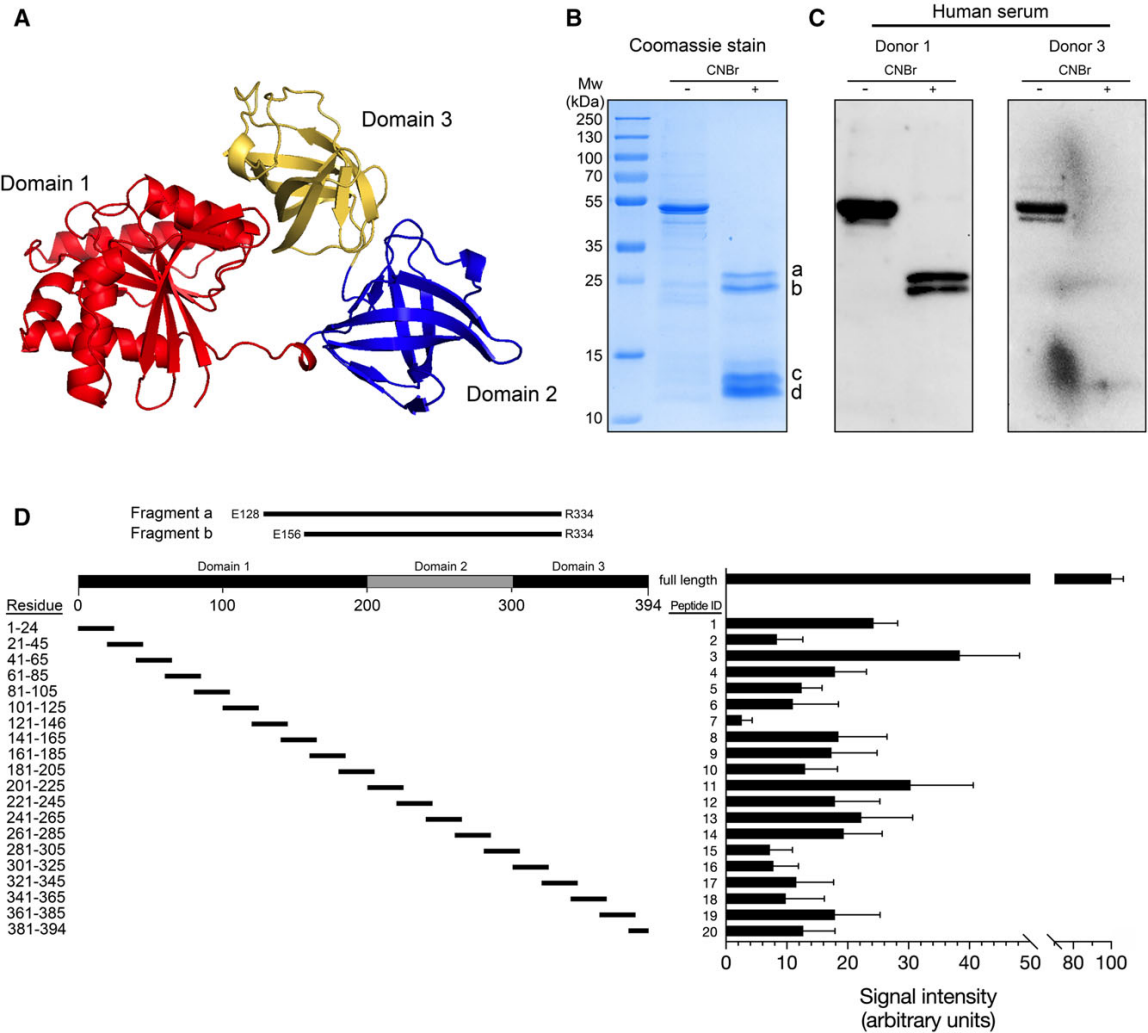
Recognition of EF‐Tu domains by human IgG. (A) Three‐dimensional structure of EF‐Tu, based on PDB 1dg1 38, 39, showing the organization of EF‐Tu domains 1‐3. (B) Recombinant NTHi EF‐Tu was analyzed by SDS‐PAGE to determine the fragmentation pattern from CNBr cleavage assay. The indicated cleaved bands a and b were in‐gel trypsin digested, excised, and sequenced to identify the corresponding fragments depicted in (D). Fragments a and b represent two products of EF‐Tu cleavage by CNBr. (C) Western blots of the CNBr‐digested EF‐Tu with two different human sera positive for IgG against full‐length EF‐Tu are shown. (D) Reactivity of adult human sera (*n = *8) positive for IgG against full‐length EF‐Tu against a set of synthetic peptides spanning the NTHi EF‐Tu sequence using dot blot. IgG reactivity against full length recombinant EF‐Tu was set as 100 arbitrary units, and mean values obtained by scanning densitometry are shown. Only sera (*n = *8) that contained high anti‐EF‐Tu IgG concentrations in ELISA (Fig. 1) were selected for dot blot analysis against EF‐Tu peptides. The experiment was performed once with sera from 8 different individuals. Data are shown as mean density from 8 donors ± SEM.

NTHi EF‐Tu consists of three structural domains, the GTP‐binding domain 1 and two oligonucleotide binding domains (domains 2 and 3) 21 (Fig. 3A). To analyze what parts of the EF‐Tu molecule that were recognized by human IgG, recombinant NTHi EF‐Tu purified from *E. coli* was subjected to chemical cleavage by cyanogen bromide (CNBr). This procedure generated four larger fragments (denoted a–d in Fig. 3B). These fragments, in addition to full length recombinant EF‐Tu, were thereafter subjected to immunoblotting with human serum samples that were chosen from those that bound most full‐length EF‐Tu in ELISA (Fig. 1). Human sera recognized full‐length EF‐Tu in the Western blot (Fig. 3C), and serum from one donor readily detected the larger EF‐Tu fragments a and b (Fig. 3C; Donor 1).

To further investigate the specificity of the response, we designed a series of peptides (25 residues long) spanning the entire EF‐Tu sequence (Fig. 3D, Fig. S1). EF‐Tu peptides were subjected to human serum using several sets of dot blots. A clear signal could be observed for most peptides, including peptides covering fragments a and b (Fig. 3D). However, the lower signals obtained with specific peptides when compared to full length EF‐Tu indicated that native protein folding may be required for optimal binding. In conclusion, human antibodies detect full‐length recombinant EF‐Tu, and in particular, the central and C‐terminal parts of the molecule.

### Mouse anti‐EF‐Tu serum is bactericidal against NTHi

**Figure 4:**
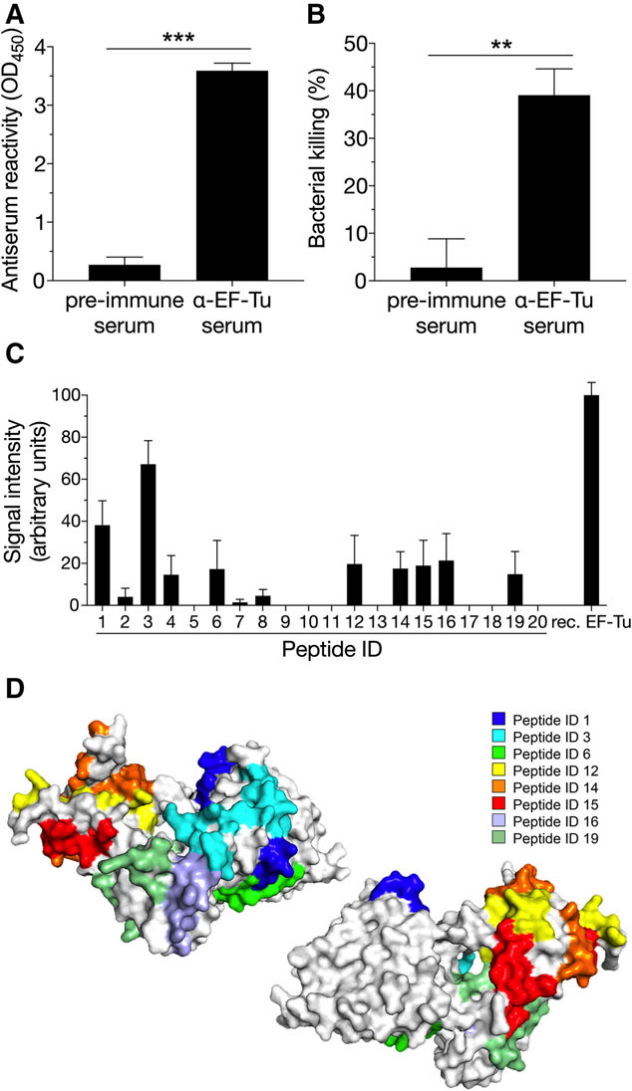
Targeting of EF‐Tu epitopes by anti‐EF‐Tu pAbs produced in mice exposed to recombinant protein. (A) Mean values of serum reactivity against recombinant EF‐Tu are shown for BALB/c (*n = *4) and C57BL/6 (*n = *4) mice immunized with recombinant EF‐Tu. Sera from non‐immunized mice were used as controls (*n = *4). (B) Serum bactericidal activity (SBA) assay with serum from mice immunized with whole EF‐Tu. Bacterial killing represents differences in CFU count after incubation with active or heat‐inactivated baby rabbit complement. (C) The reactivity of whole sera from mice (*n = *4) immunized with recombinant EF‐Tu tested against synthetic EF‐Tu peptides and full‐length EF‐Tu using dot blot. IgG reactivity against full length recombinant EF‐Tu was set as 100 arbitrary units, and mean values obtained by scanning densitometry are shown. In (A), sera from eight immunized mice (BALB/c and C57BL/6) were analyzed in technical triplicates, and data are shown as mean ± SEM for three or more independent experiments. In (B), data are shown as mean ± SEM based on five independent experiments with pooled sera (pre‐immune sera or sera from EF‐Tu immunized mice) derived from in total five different mice. (C) Data are shown as mean ± SEM from four independent sera obtained from mice that were immunized with recombinant EF‐Tu. The dot blot was done once, and error bars indicate the variation (SEM) between different mice (*n = *4) immunized with EF‐Tu. ***p < 0.001; **p < 0.01. Statistical significance was calculated using the Mann–Whitney test. (D) Schematic map of the EF‐Tu crystal structure (Protein Data Bank entry 1dg1). Peptides ID 1, 3, 6, 12, 14, 15, 16, and 19 recognized by mouse serum are indicated in the figure.

To characterize the adaptive immune response against recombinant EF‐Tu, we collected sera from immunized mice 2 weeks after administration of the last dosage. Serum reactivity against EF‐Tu was determined by ELISA (Fig. 4A), and a significant response was seen with sera from immunized mice when compared to non‐immunized animals (pre‐immune sera). To determine the functionality of the immune response, we performed a series of serum bactericidal activity (SBA) assays, and hence, analysis of the classical pathway of the complement system (Fig. 4B). NTHi were incubated with EF‐Tu antiserum or serum from non‐immunized mice in the presence of active baby rabbit complement. A significant increase in bacterial killing was observed with EF‐Tu antiserum, when compared to controls. Approximately 40% of bacteria were killed when incubated with the antiserum, whereas less than 3% was killed by pre‐immune (naïve) mouse serum supplemented with baby rabbit complement.

To pin‐point the IgG‐dependent target sequence(s) of EF‐Tu, we analyzed mouse sera against EF‐Tu peptides (outlined in Fig. 3D) by dot blot (Fig. 4C). The strongest response was seen with peptide ID 3 corresponding to sequence EF‐Tu^41‐65^. In addition to peptide ID 3, there was a cluster of immunogenic peptides (peptide ID 12‐16) approximately corresponding to the CNBr fragments a and b (Fig. 3D). This epitope pattern was confirmed by modeling the EF‐Tu surface structure showing that the immunogenic epitopes recognized by mouse sera were indeed surface exposed (Fig. 4D). In conclusion, our data demonstrate that EF‐Tu is immunogenic in the mouse, the IgGs produced are effective in promoting bacterial killing, and, finally, mouse antisera recognize approximately the same epitopes on the EF‐Tu molecule as human sera.

### EF‐Tu immunization partially protects mice from NTHi infection in the nasopharynx and middle ear

**Figure 5:**
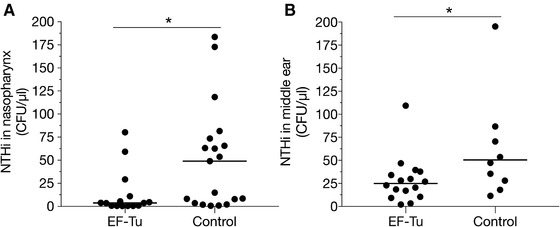
An increased clearance of NTHi is observed in mice immunized with recombinant EF‐Tu. (A) Balb/c mice (*n = *38) were immunized with recombinant full‐length EF‐Tu (*n = *17) or BSA (*n = *21). Two weeks after immunization mice were challenged with 10^8^ CFU NTHi 3655 intranasally for 20 h. Bacterial load is shown as bacterial CFU recovered per microliter of nasopharynx homogenate. Each filled circle represents the recovered CFU per microliter of nasopharynx homogenate from each mouse. (B) Junbo mice immunized with recombinant full‐length NTHi EF‐Tu (*n = *11) or with a recombinant control protein (*n = *7) were then infected with streptomycin‐resistant NTHi 3655. Colonization rates 6 d post‐infection are shown as bacterial CFU recovered per microliter of middle ear bulla fluid. Each filled circle represents the recovered CFU per microliter middle ear fluid from each mouse ear (two ears per animal), (*n = *16 [EF‐Tu immunized goup]; *n = *10 [control group]). Of note, three and two animals from EF‐Tu and control groups respectively, were excluded from enumeration due to resistance to experimental infection. The horizontal bars indicate the median values of each data set. Each panel represents one experiment with a total of 38 and 18 mice in (A) and (B), respectively. Experiments in (A) and (B) were done in separate laboratories and immunization series were performed separately at different occasions in Sweden and the UK, respectively. *p < 0.05. Statistical significance was calculated using Mann–Whitney test.

To assess whether immunization with recombinant EF‐Tu produced in *E. coli* is protective against NTHi infection in the mouse, we selected two different mouse models. First nasopharyngeal clearance in BALB/c mice was analyzed and thereafter the effect on otitis media in the otitis‐prone Junbo mice (C3H/HeH) was determined 22. BALB/c mice were immunized with four doses of full‐length recombinant NTHi EF‐Tu. Two weeks after the last immunization, mice were challenged with 10^8^ NTHi colony‐forming units (CFU) intranasally for 20 h. Nasopharyngeal bacterial load was thereafter assessed by counting CFU (Fig. 5A). Interestingly, mice immunized with EF‐Tu harbored significantly less bacteria in the nasopharynx compared to control mice immunized with bovine serum albumin (BSA).

The Junbo mouse model has a mutation in the transcription factor Evi1, causing it to develop spontaneous otitis media 23. Junbo mice were immunized with three doses of full‐length recombinant EF‐Tu and challenged with streptomycin‐resistant NTHi two weeks after the last immunization. Bacteria in the middle ear bulla fluid were enumerated by counting CFU 6 days post‐infection. In parallel with the nasopharyngeal clearance in BALB/c, partial protection against NTHi infection was seen in Junbo mice immunized with recombinant EF‐Tu as compared to animals that received a recombinant control protein (Fig. 5B).

## Discussion

Here. we show that IgG Abs directed against EF‐Tu are present in children, and that titers increase in an age‐dependent manner. In addition, infection with NTHi was associated with more EF‐Tu specific antibodies. The importance of EF‐Tu IgG for protection against NTHi was further proven by immunization of mice with recombinant EF‐Tu. Mouse convalescent serum recognized similar parts of the EF‐Tu molecule as human serum, and this serum mediated direct killing of NTHi in vitro by initiating the classical pathway of the complement system. Our data thus suggest that anti‐EF‐Tu IgGs may play an important role in the adaptive and innate immune response against NTHi.

The anti‐EF‐Tu IgG detected in children (Figs. 1 and 2A) is likely associated with previous infection and/or colonization with NTHi, but does not exclude that other potentially harmful bacteria, including *Moraxella catarrhalis* and pneumococci may also trigger the adaptive immune system, resulting in increased production of anti‐EF‐Tu IgG. The age‐dependent increase in anti‐EF‐Tu IgG (Figs. 1 and 2A) suggests a continuous exposure to the EF‐Tu antigen via different infection episodes throughout life. This assumption argues against the contribution of the microbiome (normal flora) in triggering an anti‐EF‐Tu IgG response, which is usually present already a few days after birth 24. We thus hypothesize that childhood exposure to respiratory pathogens such as NTHi induces the adaptive immune response with production of IgG against EF‐Tu. This was evident among children with a current episode of AOM 25, 26, whom had significantly more IgG against EF‐Tu than age‐matched controls (Fig. 2B and C). The cross‐reactivity induced by commensal bacteria and their possible contribution to an early anti‐EF‐Tu IgG increase must, however, be further investigated.

There is a medical need for a vaccine against NTHi, in particular for preschool children and patients suffering from COPD. Several vaccine candidates, including outer membrane lipoprotein e (P4), peptidoglycan‐associated lipoprotein P6, type IV pili protein PilA, and Protein D, E, and F, have been identified in recent years 9, 10, 11, 27, 28. A prerequisite for a potential vaccine is an adequate antibody response upon immunization resulting in functional antibodies. Here, we show that mice immunized with recombinant NTHi EF‐Tu produce antibodies that trigger the classical pathway of the complement system (Fig. 4B), and thus aid in killing of NTHi. Our data support the previous findings that EF‐Tu from *Mycoplasma ovipneumoniae* and *Neisseria gonorrhoeae* is immunogenic 29, 30 and elicits a significant immune response in mice. These results are also consistent with previous work that has shown that antisera from mice immunized with recombinant EF‐Tu achieved significant growth inhibition in vitro of the sheep pathogen *M. ovipneumoniae* 29.

An important observation is that immunization of BALB/c and Junbo (C3H/HeH) mice with recombinant EF‐Tu resulted in a partial protection against NTHi colonization in the nasopharynx and middle ear infection, respectively (Fig. 5A and B). In fact, EF‐Tu has more or less indirectly been included in various immunization protocols. Proteomic profiling of a *Neisseria meningitidis* outer membrane vesicle (OMV)‐based vaccine used to combat serogroup B outbreaks revealed EF‐Tu content within the vesicle 31. In recent attempts of intravaginal immunization of mice with *N. gonorrhoeae*‐derived OMVs, EF‐Tu was found to be the immunodominant antigen within the OMVs. These mice were also able to clear a gonococcal infection in significantly less time 30. Furthermore, in search for a vaccine against *Burkholderia pseudomallei*, immunization with recombinant EF‐Tu reduced pulmonary bacterial load in mice when challenged with the proxy *B. thailandensis* 32. The authors suggested that a Th1 response is elicited by immunization with EF‐Tu, which represents a feasible antigen for protection against meliodiosis. In parallel, we found that mice immunized with EF‐Tu produced significant titers of anti‐EF‐Tu polyclonal Abs (Fig. 4A), and that these antibodies were effective in triggering the classical pathway of complement activation (Fig. 4B). This observation may suggest a Th2 dominance in the response to immunization with EF‐Tu, but needs further verification. Our results collectively suggest that EF‐Tu may be included in a multicomponent vaccine together with other immunogenic proteins such as Protein D 33 and Protein F 10, 34. However, the close homology in EF‐Tu derived from different bacterial species poses a potential threat to the normal flora (microbiota). Another potential worry is the archaebacterial origin of mitochondria and the similarities of mitochondrial EF‐Tu to bacterial EF‐Tu (48% identity with NTHi EF‐Tu). We, however, observed no such cross‐reactivity between anti‐EF‐Tu‐positive human serum (IgG) and mitochondrial EF‐Tu in ELISA (data not shown). The full extent of cross‐reactivity after immunization must most likely be determined before EF‐Tu can be considered a viable vaccine antigen.

In conclusion, we have shown that IgG antibodies against NTHi EF‐Tu are present in humans at an early stage and increase with age. Antibody titers were also seen to correlate with exposure to NTHi infection, and the immunodominant epitopes in the human immune response was found to reside primarily at the C‐terminal half of the molecule. Immunization of mice with recombinant NTHi EF‐Tu triggered a significant production of functional antibodies and provided protection against NTHi. Further studies are needed to fully elucidate the role of surface‐associated NTHi EF‐Tu in bacterial fitness and the relevance of this moonlighting protein as a vaccine candidate in a future vaccine aimed at NTHi.

## Materials and methods

### Bacteria and culture conditions

The NTHi 3655 was a gift from R. Munson (The Ohio State University, Columbus, OH) 35. NTHi strain 3655 expressing resistance to streptomycin was generated by selecting spontaneous streptomycin‐resistant colonies after plating bacteria at high density (2 × 10^10^ CFU per plate) on media containing streptomycin (300 μg/mL). NTHi wild‐type and mutants were routinely cultured in brain–heart infusion (BHI) liquid broth supplemented with NAD and hemin (both at 2 μg/mL) or on chocolate agar plates at 37 °C in a humid atmosphere containing 5% CO_2_.

### Production of recombinant NTHi EF‐Tu and EF‐Tu peptides

The open reading frame (ORF) of the gene encoding full‐length NTHi EF‐Tu (EDJ92442.1) was amplified from NTHi 3655 genomic DNA using the primer pair 5′‐GGGGCGGATCCGATGTCTAAAGAAAAATTTGAACGTA‐3′/5′‐GGCGGAAGCTTTTTGATGATTTTCGCAACAACGCCA‐3′ containing restriction enzyme sites *Bam*HI and *Hin*dIII (underlined), respectively. Following restriction enzyme digestion, the resulting DNA fragment (1214 base pairs) was cloned into the expression vector pET26(b)+ (Novagen Merck, Darmstadt, Germany) for recombinant protein production as described previously 9. Briefly, the resulting plasmid was transformed into *E. coli* DH5α, followed by DNA sequencing. Recombinant proteins were thereafter produced in *E. coli* BL21 (DE3) and purified by affinity chromatography using Ni‐NTA agarose. To exclude that trace amounts of *E. coli* lipopolysaccharide (LPS) in the recombinant EF‐Tu preparations did not cause a background in ELISAs, human and mouse sera were tested against purified LPS derived from *E. coli* (Sigma–Aldrich, St Louis, MO) in ELISA. LPS was titrated and only a low IgG reactivity was observed against LPS at 100 and 500 ng/well in anti‐EF‐Tu positive human sera diluted 1/100, whereas no reactivity was seen with 1 or 10 ng LPS/well. This was in sharp contrast to anti‐EF‐Tu IgG reactivity, and consequently a background titer caused by anti‐LPS IgG Abs was excluded. In parallel, sera from immunized mice showed no reactivity against LPS in the same concentration range. The recombinant EF‐Tu contained trace amounts of LPS (0.3 ng/mL corresponding to 0.06 ng/well in ELISA) according to a Limulus assay (Associates of Cape Cod, East Falmouth, MA). For NTHi epitope mapping, 20‐ to 25‐residue‐long peptides, overlapping by five residues and together covering the entire EF‐Tu sequence (Table S1), were synthesized by Genscript (Piscataway, NJ).

### Antisera and antibody preparation

Mouse anti‐EF‐Tu sera were prepared as previously described 10. Briefly, BALB/c and C57BL/6 mice were immunized intraperitoneally with 50 μg of EF‐Tu in 100 μl saline and 100 μl Freund's adjuvant. Animals were boosted 3 times every 4 weeks with alum as adjuvant and blood was drawn 2 weeks after the last immunization. Polyclonal antibodies were affinity purified using EF‐Tu coupled to CNBr‐activated SepharoseTM (GE Healthcare Biosciences, Chicago, IL). Control mice were immunized with either BSA (nasopharyngeal carriage) or a recombinant NTHi protein (hypothetical protein, GenBank: EDJ92307) that is not surface‐exposed.

### Human serum samples

Plasma samples from children (*n = *109), sampled at 18 or 36 months and 6 years of age, were obtained from Sahlgrenska University Hospital (Gothenburg, Sweden). Adult blood donor sera (*n = *72) were from Clinical Microbiology (Laboratory Medicine, Malmö, Sweden). In addition, children plasma samples (*n = *195) collected under NIH‐R01‐DC008671 from Rochester, New York area were analyzed for anti‐EF‐Tu antibodies; sample collection and microbial identification has been described in detail previously (28). Donor information was kept anonymous after sampling.

### Enzyme linked immunosorbent assay (ELISA)

Following the addition of 0.5 μg of purified recombinant EF‐Tu, Protein D 33, or Protein F 9 in 0.1 M Tris (pH 9) per well, Nunc PolySorp 96‐well microtiter plates (Thermo Fisher Scientific, Waltham, MA) were incubated overnight at 4°C. After four washes, the plates were blocked for 1 h at room temperature (RT) with 250 μL PBS with 0.05% Tween 20 (PBST) with 1% BSA per well. After washing, the samples were incubated for 1 h at RT with sera diluted 1:100 in PBST with 2.5% BSA and subsequently washed three times. Horseradish peroxidase (HRP)‐conjugated rabbit anti‐human pAbs (Dako, Glostrup, Denmark) were added for 1 h at RT, with four washes following the incubation. At all steps, each wash was performed for 5 min using PBST. Antibody‐antigen complexes were detected using the hydrogen peroxide/3,3′,5,5′‐tetramethylbenzidine (TMB) substrate solution, with reactions stopped with 1 M sulfuric acid, followed by determination of the absorbance at 450 nm. For detection of anti‐EF‐Tu antibodies (IgG) in children, a similar methodology was used except blocking was done with 3% skim milk and plasma samples were run at a starting dilution of 1:200 and serially diluted to 1:25600, and allowed to incubate for 1.5 h at RT. On each plate a standard was included using capture IgG as the coating antigen and a human reference serum with a known concentration of IgG to use for comparison in determining IgG titers of unknown children samples. The secondary antibody used was goat anti‐human IgG conjugated with alkaline phosphatase (AP), at 1:5000 concentration at RT for 1 h and the reaction was stopped with 3 M NaOH before plates were read on SPECTRAmax PC340 reader at 405 nm, and absorbance data imported into SoftMax Pro 5.4 where raw data were processed to determine IgG antibody titers for each sample in μg/mL. For assessment of anti‐EF‐Tu reactivity in mouse serum the same procedure was used, but plates were coated with 1 μg recombinant EF‐Tu, and HRP‐conjugated rabbit anti‐mouse pAbs (Dako) used as secondary antibody.

### Structural modeling

Modeling of NTHi EF‐Tu was performed using the Swiss‐Model automated server against homologous templates available in the Protein Data Bank (PDB; available at: http://www.rcsb.org). Three‐dimensional models were prepared using PyMOL (available at: http://www.pymol.org/).

### SDS‐PAGE and Western blotting

Samples with full‐length EF‐Tu or CNBr‐digested EF‐Tu were separated on a 12% polyacrylamide gel and either stained with Coomassie Brilliant Blue R‐250 (Bio‐Rad, Munich, Germany) or transferred onto a 0.45 μm Immobilon‐P PVDF Membrane (Millipore, Bedford, MA) at 16 V for 15 h. Following blocking in 5% skim milk in PBS, membranes were incubated at RT for 1 h with human sera diluted 1:100 in 5 mL PBS with 5% skim milk. Following three washes in PBS, the membranes were incubated for 1 h with HRP‐conjugated goat anti‐human pAb (Dako). The membranes were thereafter washed in PBST, developed using Pierce™ Enhanced Chemiluminescence (ECL) Western blotting substrate (Thermo Fisher Sientific), and visualized on a BioRad ChemiDoc™.

### Peptide‐based epitope mapping

To identify the epitopes recognized by the anti‐EF‐Tu IgG, EF‐Tu peptides (20 μg; Peptide ID 1‐20; Fig. 4B and Fig. S1) or full‐length EF‐Tu (0.05, 0.5, and 5 μg) were coated onto nitrocellulose (0.45 μm pore size) (NC) membranes. The membranes were dried for 30 min at 37°C, stained with Ponceuau S, and thereafter blocked for 1 h at RT in PBST with 1% BSA and 1% casein. The membranes were therefter incubated overnight at 4°C with sera diluted 1:100 in PBST with 1% BSA and 1% casein. The membranes were incubated with HRP‐conjugated rabbit anti‐human or rabbit anti‐mouse pAbs (Dako) for 20 min at RT, with four 10 min washes in PBST performed prior to and following the incubation. Membranes were developed using PierceTM ECL Western Blot substrate and visualized on a BioRad ChemiDocTM. Pixel density of the dot blot images was assessed using ImageJ^®^ version 1.51.

### Serum bactericidal activity (SBA) assay

Susceptibility of antibody‐exposed bacterial cells to complement‐mediated killing was assessed using the SBA assay as previously described 9. Briefly, 4 × 10^4^ CFU of NTHi 3655 was resuspended in 5 μL Hank's balanced salt solution with 2% heat‐inactivated baby rabbit complement (Nordic BioSite AB, Täby, Sweden) and 400 mM d‐mannose, followed by incubation with 20 μL EF‐Tu mouse antiserum or preimmune mouse serum for 30 min at RT. d‐Mannose was added to block mannose‐binding lectins and thus the lectin pathway of the complement system 36. After the addition of active or heat‐inactivated baby rabbit complement to a final concentration of 2.5%, the samples were incubated for 1 h at 37°C with gentle shaking. Aliquots (10 μL) from the reaction mixtures were plated on chocolate agar, and CFUs were determined after overnight incubation.

### Nasopharyngeal clearance mouse model

Female BALB/c mice at 8 weeks of age were immunized as described above and challenged with 10^8^ CFU NTHi intranasally for 20 h. Mice were euthanized, the nasopharynx removed, homogenized, and mixed with BHI broth supplemented with 10 μg/mL NAD (Sigma–Aldrich) and 10 μg/mL hemin (Merck). The mixture was then plated on chocolate agar plates and enumeration of colonies used to calculate nasopharyngeal infection rates.

### Otitis media (OM) mouse model

Heterozygote Junbo mice (C3H/HeH) spontaneously display symptoms of chronic OM, including a thickened tympanic membrane and increased middle ear fluid, due to a mutation in the Evi1 gene. Lack of other organ pathology or overt immune deficiency qualifies these mice as a suitable model for human OM 22, 37. Briefly, 8–10 week‐old Junbo mice were immunized with three doses of recombinant full‐length NTHi EF‐Tu (*n = *16) or with a recombinant control protein (*n = *10). Two weeks after the last immunization, mice were inoculated intranasally with 10^6^ CFU of streptomycin‐resistant NTHi. After 6 days, middle ear fluid samples were collected and plated on BHI agar containing streptomycin, followed by CFU enumeration to calculate middle ear bacterial load.

### Ethics statements

Anonymized normal human sera (*n = *72) were obtained from healthy adult blood donors at Skane University Hospital, Malmo, Sweden undergoing screening for blood contamination. This sampling was according to a general IRB approval for Division of Laboratory Medicine Skåne (Region Skåne, Sweden). For the collection of sera from children in Gothenburg (*n = *204) and Rochester (*n = *135), written informed consent was obtained from all parents prior to sampling. The IRBs of the University of Rochester and the Rochester Regional Health System approved the study. The study in Sweden was approved by the IRB and Research Ethics Committee of the Medical Faculty, University of Gothenburg (446‐00, approved 2000‐09‐19 and 362‐2005, approved 2005‐08‐01). Ethical permits for animal experiments (M106‐16, M107‐16, M193‐11, M321‐12) were obtained from Malmö/Lund District Court (Djurförsöksetiska nämnden, Tingsrätten, Byggmästaregatan 2, SE‐222 37 Lund, Sweden). Experiments with Junbo mice were carried out under the authority of the appropriate UK Home Office Project License and were approved by the Medical Research Council Harwell ethical review committee.

### Statistics

Student´s *t*‐test was used for analysis of parametrical sets of data and the Mann–Whitney *U* test was used for nonparametric data sets. One‐way ANOVA was used when comparing two or more experimental groups, with Bonferroni post hoc test. Differences were considered statistically significant at *p* ≤ 0.05. All analyses were performed using GraphPad Prism^®^ version 7.0 (GraphPad Software, La Jolla, CA).

## Author Contributions

Experiment Design: M.B., D.H., R.K., K.R., A.R., O.T., and Y‐C.S. Performed experiment: M.B., D.H., R.K., O.T., and Y‐C.S. Wrote and Edited the Manuscript: M.B., D.H., R.K., K.R., A.R., O.T., and Y‐C.S.

## Conflict of interest

The authors declared no conflict of interest.

**Abbreviations**
AOM: acute otitis media
BHI: brain‐heart infusion
CFU: colony‐forming units
CNBr: cyanogen bromide
EF‐Tu: elongation‐factor thermo unstable
NTHi: non‐typeable *Haemophilus influenzae*
OMV: outer membrane vesicle
PBST: PBS with 0.05% Tween 20
RT: room temperature
SBA: serum bactericidal activity

## Supporting information

Peer review correspondence

Supporting Information
