# Supplementary material for: Anti‐EF‐Tu IgG titers increase with age and may contribute to protection against the respiratory pathogen Haemophilus influenzae
Source: Eur J Immunol. 2019 Jan 3;49(3):490–9. doi: 10.1002/eji.201847871 (PMC6491980; doi:10.1002/eji.201847871)
Supplement: Supplementary file 2 — Supporting Information [file EJI-49-490-s002.pdf]

European Journal of Immunology

Supporting Information
for
DOI 10.1002/eji.201847871

Oskar Thofte, Ravinder Kaur, Yu-Ching Su, Marta Brant, Anna Rudin, Derek Hood and Kristian Riesbeck

Anti-EF-Tu IgG titers increase with age and may contribute to protection against the respiratory pathogen *Haemophilus influenzae*

© 2018 The Authors. *European Journal of Immunology* published by WILEY-VCH Verlag GmbH & Co. KGaA, Weinheim.

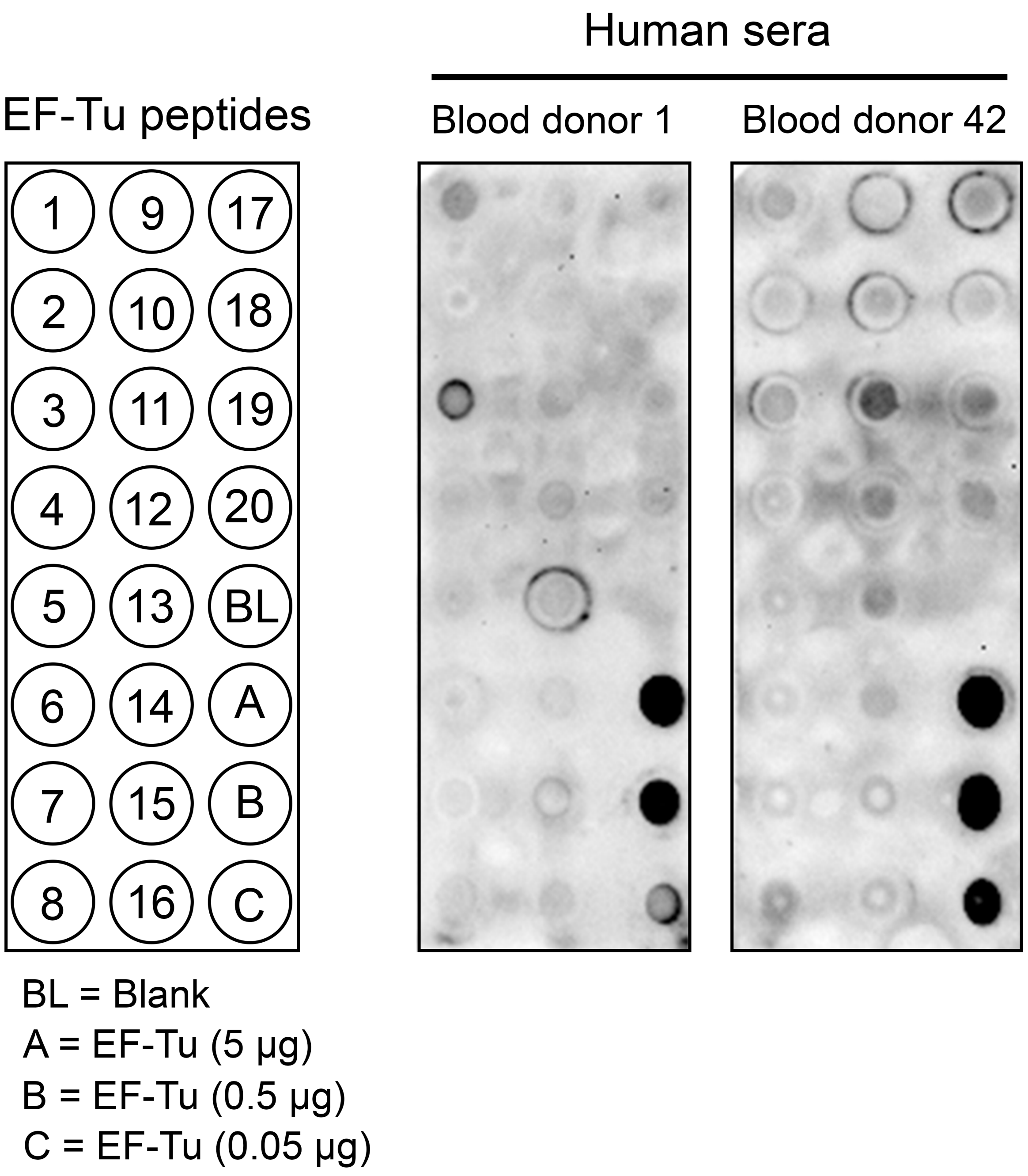

Figure S1. An example of peptide mapping of EF-Tu immunodominant epitopes recognized by human serum. Epitope mapping was performed using dot blots of EF-Tu peptides probed with serum from human blood donors. Human sera were incubated with filters containing spotted peptides, followed by incubation with HRP-conjugated anti-human IgG antibodies. Semi quantitative analysis of detected spot signal densities was performed and is shown in Figure 3D.

Table S1. Peptide library of EF-Tu (NTHi 3655)

	Peptide ID	Sequence	Number of residues
EF-Tu$^{1-25}$	1	MSKEKFERTKPHVNVGTIGHVDHGK	25
EF-Tu$^{21-45}$	2	VDHGKTTLTAAITTVLAKHYGGAAR	25
EF-Tu$^{41-65}$	3	GGAARAFDQIDNAPEEKARGITINT	25
EF-Tu$^{61-85}$	4	ITINTSHVEYDTPTRHYAHVDCPGH	25
EF-Tu$^{81-105}$	5	DCPGHADYVKNMITGAAQMDGAILV	25
EF-Tu$^{101-125}$	6	GAILVVAATDGPMPQTREHILLGRQ	25
EF-Tu$^{121-146}$	7	LLGRQVGVPYIIVFLNKCDMVDDEE	25
EF-Tu$^{141-165}$	8	VDDEELLELVEMEVRELLSQYDFPG	25
EF-Tu$^{161-185}$	9	YDFPGDDTPIVRGSALQALNGVAEW	25
EF-Tu$^{181-205}$	10	GVAEWEEKILELAGHLDTYIPEPER	25
EF-Tu$^{201-225}$	11	PEPERAIDQPFLLPIEDVFSISGRG	25
EF-Tu$^{221-245}$	12	ISGRGTVVTGRVERGIIRTGDEVEI	25
EF-Tu$^{241-265}$	13	DEVEIVGIKDTAKTTVTGVEMFRKL	25
EF-Tu$^{261-285}$	14	MFRKLLDEGRAGENIGALLRGTKRE	25
EF-Tu$^{281-305}$	15	GTKREEIERGQVLAKPGSITPHTDF	25
EF-Tu$^{301-325}$	16	PHTDFESEVYVLSKDEGGRHTPFFK	25
EF-Tu$^{321-345}$	17	TPFFKGYRPQFYFRTTDVTGTIELP	25
EF-Tu$^{341-365}$	18	TIELPEGVEMVMPGDNIKMTVSLIH	25
EF-Tu$^{361-385}$	19	VSLIHPIAMDQGLRFAIREGGRTVG	25
EF-Tu$^{381-394}$	20	GRTVGAGVVAKIIK	14
