# Supplementary material for: Anti‐EF‐Tu IgG titers increase with age and may contribute to protection against the respiratory pathogen Haemophilus influenzae
Source: Eur J Immunol. 2019 Jan 3;49(3):490–9. doi: 10.1002/eji.201847871 (PMC6491980; doi:10.1002/eji.201847871)
Supplement: Supplementary file 1 — Peer review correspondence [file EJI-49-490-s001.pdf]

European Journal of Immunology

Anti-EF-Tu IgG titers increase with age and may contribute to protection against the respiratory pathogen *Haemophilus influenzae*

Thofte, Oskar; Kaur, Ravinder; Su, Yu-Ching; Brant, Marta; Rudin, Anna; Hood, Derek; Riesbeck, Kristian

Review Timeline:	Submission date:	18-Aug-2018
	First Editorial decision:	18-Sep-2018
	Revision/s received:	23-Nov-2018
	Accepted:	17-Dec-2018

Handling Executive Committee member: Prof. Annette Oxenius

Please note that the correspondence below does not include the standard editorial instructions regarding preparation and submission of revised manuscripts, only the scientific revisions requested and addressed.

First Editorial Decision - 18-Sep-2018

Dear Prof. Riesbeck,

Manuscript ID eji.201847871 entitled "Increased anti-EF-Tu IgG titers with age mediate protection against the respiratory pathogen Haemophilus influenzae", which you submitted to the European Journal of Immunology, has been reviewed. The comments of the referees are included at the bottom of this letter.

Although the referees have recommended publication, some revisions to your manuscript have been requested. Therefore, I invite you to respond to the comments of the referees and revise your manuscript accordingly.

You should also pay close attention to the editorial comments included below. **In particular, please edit your figure legends to follow Journal standards as outlined in the editorial comments. Please clearly state the number of independent experiments performed. Failure to do this will result in delays in the re-review process.**

If the revision of the paper is expected to take more than three months, please inform the editorial office. Revisions taking longer than six months may be assessed by new referee(s) to ensure the relevance and timeliness of the data.

Once again, thank you for submitting your manuscript to European Journal of Immunology. We look forward to receiving your revision.

Yours sincerely,
Laura Soto Vazquez

on behalf of Prof. Annette Oxenius

Editorial Office
European Journal of Immunology
e-mail: ejied@wiley.com
www.eji-journal.eu

****

Reviewer: 1
Comments to the Author

Thofte et al report elevated titres of anti-EF-tu antibodies with Age and during episodes of NTHi Otitis media in humans. The correlate this with mouse Experiments that suggest a small functional effect of these antibodies.

Minor revisions.
1. Statistics:fig. 1 shows a time series. If you are comparing time and antibody Targets then a 2-way anova would be more appropriate.
In all figures, as the data is unlikely to be truly normally distributed, it would be more appropriate to plot medians and ranges than mean and standard Deviation. The poor representative power of the mean is particularly clear in Figure 5 (strongly influenced by the few high values)
2. With regard to the high conservation of EF-Tu between bacterial species, could the authors comment on the degree of homology to mitochondrial EF-Tu? Particularly to peptides 1 and 3 that are more strongly recognised in the mouse vaccination studies? If high homology exists, the potential for autoimmunity and the absence of central tolerance should be briefly discussed.
3. The antibody binding to peptides, particularly with the human sera, is dramatically lower than binding to the whole Protein. This rather suggests that the epitope may require native Protein folding, and makes the peptide scanning difficult to Interpret. This should be briefly commented in the results.
4. The arguementation for why this cannot be driven by commensal microbes is somewhat unclear. The microbiota in certainly present from shortly after birth, but given the low Level of invasiveness, antibody

Responses may develop only slowly to These bacteria. One could compare germ-free and colonized mice, but I feel this is not central to the message of the paper and therefore not necessary. It would be wiser to simply comment that a contribution of the microbiota is possible
5. Title: given the short-comings of the mouse models, "mediate protection" is a bit strong. How about "Anti-EF-Tu IgG titers increase with age and can contribute to protection against the respiratory pathogen Haemophilus influenzae"?

Reviewer: 2
Comments to the Author

Thofte et al., report that NTHi EF-Tu is a target of specific antibodies in children and adults and that the titres of these antibodies increase with age. Antibodies to NTHi EF-Tu are also increased in patients with AOM who are infected with NTHi. NTHi EF-Tu is immunogenic in mice inducing bactericidal antibodies and reduction of bacterial load in nasopharynx and middle ear in mice challenged after 4 doses of 50 microgram of EF-Tu mixed with adjuvants. Epitopes mapping using human and mice antibodies showed similar targets within the EF-Tu.
I did not find previous reports on the antigenicity and immunogenicity of NTHi EF-Tu. I consider is relevant to identify molecules involved in the onset of immunity against NTHi that could lead to correlates of protection, to the better understanding of how immunity against this disease is achieved and for vaccine development. The manuscript is well presented, clearly explained and data supports authors' interpretations.

Minor comments:
1. Please indicate the LPS content in the EF-Tu used for immunisation and for ELISA test and add a control showing antibody titres observed in humans and mice are not due to the LPS contamination.
2. T cell responses to EF-Tu is not reported here, if you have the data please add it to the manuscript. In the discussion is said that Th2 responses is most likely achieved by NTHi EF-Tu, however data does not support this conclusion. IgG isotypes have not been measured, neither T cell responses nor cytokines and markers, to support this statement. Both Th1 and Th2 can promote antibody responses. In particular, immunisations with bacterial derived antigens often induce different T cell subsets.
3. “The anti-EF-Tu IgG detected in children (Fig. 1 and 2A) is likely associated with previous infection and/or colonization with NTHi, but does not exclude that other potentially harmful bacteria”. Is there any epitope that would be NTHi EF-Tu specific? Aligning EF-Tu of different commensal or pathogens may show specific regions targeted by anti-NTHi EF-Tu antibodies, a figure and/or commentary of this analysis will be useful to understand the time increase of antibody titres during time in humans.
4. Paper could benefit with a figure showing human and mice shared epitopes.

5. Have anti-NTHi EF-Tu IgM titres measured? Please include this data and/or discuss about the role of these antibodies since other bacterial proteins such as outer membrane proteins can induce long lasting IgM antibody titres.
6. "Peptide-based epitope mapping revealed that human sera and sera from immunized mice recognized EF-Tu in a similar pattern, targeting mainly the central and C-terminal part of the molecule". However in figure 3D and 4C main signal intensity is observed in the peptides of domain 1 (amino-terminal) and domain 2. Please clarify this in the text.
7. In Mat&Meth is stated that boost doses were performed every 2-4 weeks. Please indicate the precise immunisation protocol, reference 9 leads to another reference, however the immunisation protocol there is not related to the authors description. Is also stated that blood was drawn 2 weeks after the last immunisation, however in figure 4 is stated "sera was harvested 4 weeks after last immunisation" please clarify these points in the manuscript.
8. In figure 3 and 4C is stated that the signal intensity induced by EF-Tu is consider 100%, however in the graphs, the bars reach only the 80-90% please clarify this in the graphs.
9. Please point out the groups with statistical differences in figure 1.

First Revision – authors' response 23-Nov-2018

Reviewer: 1
Comments to the Author

Thofte et al report elevated titres of anti-EF-tu antibodies with Age and during episodes of NTHi Otitis media in humans. The correlate this with mouse Experiments that suggest a small functional effect of these antibodies.

Minor revisions.
1. Statistics:fig. 1 shows a time series. If you are comparing time and antibody Targets then a 2-way anova would be more appropriate.
AU: We do not directly compare levels of antibodies against the different NTHi proteins (they are all significantly different from each other in 2-way ANOVA) but rather show development over time for each antigen, in the same figure. Hence one-way ANOVA seems appropriate in this case. We have also consulted a statistician for confirmation.

2. In all figures, as the data is unlikely to be truly normally distributed, it would be more appropriate to plot medians and ranges than mean and standard Deviation.
AU: We agree, however in Figure 1 the number of observations should be large enough to assume a

Gaussian distribution (n=89, 92, 23 and 72).

3. The poor representative power of the mean is particularly clear in Figure 5 (strongly influenced by the few high values).
AU: We thank the Reviewer for this comment. The horizontal lines in Figure 5 have been changed to represent the median of each group instead of the mean. We hope that it is clearer now.

4. With regard to the high conservation of EF-Tu between bacterial species, could the authors comment on the degree of homology to mitochondrial EF-Tu? Particularly to peptides 1 and 3 that are more strongly recognised in the mouse vaccination studies? If high homology exists, the potential for autoimmunity and the absence of central
tolerance should be briefly discussed.
AU: This is a very good comment. There is 48% identity between human mitochondrial EF-Tu and NTHi EF-Tu. We have now tested the anti-EF-Tu-positive human serum samples against mitochondrial EF-Tu in ELISA and observed no cross-reactivity. A brief comment about this has been added to the Discussion part.

5. The antibody binding to peptides, particularly with the human sera, is dramatically lower than binding to the whole Protein. This rather suggests that the epitope may require native Protein folding, and makes the peptide scanning difficult to Interpret. This should be briefly commented in the results.
AU: This is a very relevant comment, and the text has been modified accordingly in the Results section.

6. The arguementation for why this cannot be driven by commensal microbes is somewhat unclear. The microbiota in certainly present from shortly after birth, but given the low Level of invasiveness, antibody Responses may develop only slowly to These bacteria. One could compare germ-free and colonized mice, but I feel this is not central to the message of the paper and therefore not necessary. It would be wiser to simply comment that a contribution of the microbiota is possible
AU: We agree with the Reviewer that this is very puzzling, but also that this will be a matter for future studies. A sentence to conclude this has been added to the manuscript.

7. Title: given the short-comings of the mouse models, "mediate protection" is a bit strong. How about "Anti-EF-Tu IgG titers increase with age and can contribute to protection against the respiratory pathogen Haemophilus influenzae"?
AU: We agree with the Reviewer. It is correct that the effect is not optimal in the mouse models. However, mice are not optimal for testing NTHi since the bacterium is not a mouse pathogen. The Title has therefore been changed, also in accordance with the good suggestions by the Editor.

Reviewer: 2
Comments to the Author

Thofte et al., report that NTHi EF-Tu is a target of specific antibodies in children and adults and that the titres of these antibodies increase with age. Antibodies to NTHi EF-Tu are also increased in patients with AOM who are infected with NTHi. NTHi EF-Tu is immunogenic in mice inducing bactericidal antibodies and reduction of bacterial load in nasopharynx and middle ear in mice challenged after 4 doses of 50 microgram of EF-Tu mixed with adjuvants. Epitopes mapping using human and mice antibodies showed similar targets within the EF-Tu.
I did not find previous reports on the antigenicity and immunogenicity of NTHi EF-Tu. I consider is relevant to identify molecules involved in the onset of immunity against NTHi that could lead to correlates of protection, to the better understanding of how immunity against this disease is achieved and for vaccine development. The manuscript is well presented, clearly explained and data supports authors' interpretations.

Minor comments:
1. Please indicate the LPS content in the EF-Tu used for immunisation and for ELISA test and add a control showing antibody titres observed in humans and mice are not due to the LPS contamination.
AU: Reactivity against purified LPS from E. coli in positive human serum and sera from EF-Tu immunized mice has been tested and a description added to the materials and methods section. The text now reads: "To exclude that trace amounts of E. coli lipopolysaccharide (LPS) in the recombinant protein preparations did not cause a background in ELISAs, human and mouse sera were tested against purified LPS derived from E. coli (Sigma-Aldrich, St Louis, MO) in ELISA. LPS was titrated and only a low IgG reactivity was observed against LPS at 100 and 500 ng/well in anti-EF-Tu positive human sera diluted 1/100, whereas no reactivity was seen with 1 or 10 ng LPS/ well. This was in bright contrast to anti-EF-Tu IgG reactivity, and consequently a background titer caused by anti-LPS IgG Abs was excluded. In parallel, sera from immunized mice showed no reactivity against LPS in the same concentration range. The recombinant EF-Tu contained trace amounts of LPS (0.3 ng/ml corresponding to 0.06 ng/ well in ELISA) according to a Limulus assay (Associates of Cape Cod, East Falmouth, MA)." Taken together, most likely antisera and IgGs did not react with any endotoxin in our antigen preparations. Noteworthy, sterile water may have (allowed) an endotoxin content of app. 0.05 ng/ml.

2. T cell responses to EF-Tu is not reported here, if you have the data please add it to the manuscript. In the discussion is said that Th2 responses is most likely achieved by NTHi EF-Tu, however data does not support this conclusion. IgG isotypes have not been measured, neither T cell responses nor cytokines and markers, to support this statement. Both Th1 and Th2 can promote antibody responses. In particular,

immunisations with bacterial derived antigens often induce different T cell subsets.
AU: We agree with the Reviewer and accept this comment. Unfortunately, we do not have these data. Our speculation on IgG isotype has therefore been removed and wording changed considering a Th2 response.

3. "The anti-EF-Tu IgG detected in children (Fig. 1 and 2A) is likely associated with previous infection and/or colonization with NTHi, but does not exclude that other potentially harmful bacteria". Is there any epitope that would be NTHi EF-Tu specific? Aligning EF-Tu of different commensal or pathogens may show specific regions targeted by anti-NTHi EF-Tu antibodies, a figure and/or commentary of this analysis will be useful to understand the time increase of antibody titres during time in humans.
AU: This is a very interesting and important comment. However, we think that it is beyond the scope of the current manuscript. Therefore, we cite previous publications on the subject, that is, surface exposed EF-Tu on S. aureus, P. aeruginosa and S. pneumoniae among others. Please see references 16-19.

4. Paper could benefit with a figure showing human and mice shared epitopes.
AU: After studying figures 3D and 4C in great detail we think that another figure would not add much information. Due to the polyclonal pattern of the human humoral response, this figure would be very similar to Fig. 4D since all mouse epitopes are shared epitopes with the human ones.

5. Have anti-NTHi EF-Tu IgM titres measured? Please include this data and/or discuss about the role of these antibodies since other bacterial proteins such as outer membrane proteins can induce long lasting IgM antibody titres.
AU: We did not measure IgM titers, and do not have much serum left do this analysis. It is, however, a very good point and may be the impetus for further studies.

6. "Peptide-based epitope mapping revealed that human sera and sera from immunized mice recognized EF-Tu in a similar pattern, targeting mainly the central and C-terminal part of the molecule". However in figure 3D and 4C main signal intensity is observed in the peptides of domain 1 (amino-terminal) and domain 2. Please clarify this in the text.
AU: This is now clarified in the text.

7. In Mat&Meth is stated that boost doses were performed every 2-4 weeks. Please indicate the precise immunisation protocol, reference 9 leads to another reference, however the immunisation protocol there is not related to the authors description. Is also stated that blood was drawn 2 weeks after the last immunisation, however in figure 4 is stated "sera was harvested 4 weeks after last immunisation" please clarify these points in the manuscript.
AU: The reference has been changed to the correct one (reference 10) and time of serum collection has

been clarified.

8. In figure 3 and 4C is stated that the signal intensity induced by EF-Tu is consider 100%, however in the graphs, the bars reach only the 80-90% please clarify this in the graphs.
AU: We apologize for this mistake. The graphs have now been updated.

9. Please point out the groups with statistical differences in figure 1.
AU: We thank you for this comment, brackets to indicate significant differences have now been added to fig. 1.

Second Editorial Decision - 10-Dec-2018

Dear Prof. Riesbeck,

It is a pleasure to provisionally accept your manuscript entitled "Anti-EF-Tu IgG titers increase with age and may contribute to protection against the respiratory pathogen Haemophilus influenzae" for publication in the European Journal of Immunology. For final acceptance, please follow the instructions below and return the requested items as soon as possible as we cannot process your manuscript further until all items listed below are dealt with.

Please note that EJI articles are now published online a few days after final acceptance (see Accepted Articles: https://onlinelibrary.wiley.com/toc/15214141/0/ja). The files used for the Accepted Articles are the final files and information supplied by you in Manuscript Central. You should therefore check that all the information (including author names) is correct as changes will NOT be permitted until the proofs stage.

We look forward to hearing from you and thank you for submitting your manuscript to the European Journal of Immunology.

Yours sincerely,
Laura Soto Vazquez

on behalf of Prof. Annette Oxenius

Editorial Office
European Journal of Immunology
e-mail: ejied@wiley.com
www.eji-journal.eu
